# Effectiveness and Efficacy of Vaccine on Mutated SARS-CoV-2 Virus and Post Vaccination Surveillance: A Narrative Review

**DOI:** 10.3390/vaccines10010082

**Published:** 2022-01-06

**Authors:** Ihsanul Hafiz, Didi Nurhadi Illian, Okpri Meila, Ahmad Rusdan Handoyo Utomo, Arida Susilowati, Ipanna Enggar Susetya, Desrita Desrita, Gontar Alamsyah Siregar, Mohammad Basyuni

**Affiliations:** 1 Department of Pharmacology, Faculty of Pharmacy and Health, Institut Kesehatan Helvetia, Medan 20124, Indonesia; ihsanulhafiz@helvetia.ac.id; 2 Doctoral Program in Pharmacy, Department of Pharmaceutical Biology, School of Pharmacy, Institut Teknologi Bandung, Bandung 40132, Indonesia; 3 Department of Pharmacy, Faculty of Mathematics and Natural Sciences, Universitas Syiah Kuala, Banda Aceh 23111, Indonesia; illian.didinurhadi@unsyiah.ac.id (D.N.I.); okprimeila@unsyiah.ac.id (O.M.); 4 Doctoral Program of Clinical Pharmacy and Pharmacology, Faculty of Pharmacy, Universitas Indonesia, Depok 16424, Indonesia; 5 Faculty of Medicine, Universitas YARSI, Jakarta 10510, Indonesia; ahmad.rusdan@yarsi.ac.id; 6 Center of Excellence for Mangrove, Universitas Sumatera Utara, Medan 20155, Indonesia; arida.susilowati@usu.ac.id (A.S.); ipannaenggar@gmail.com (I.E.S.); desrita@usu.ac.id (D.D.); gontar@usu.ac.id (G.A.S.); 7 Department of Forestry, Faculty of Forestry, Universitas Sumatera Utara, Medan 20155, Indonesia; 8 Department of Aquatic Resource Management, Faculty of Agriculture, Universitas Sumatera Utara, Medan 20155, Indonesia; 9 Division of Gastroenterology-Hepatology, Department of Internal Medicine, Faculty of Medicine, Universitas Sumatera Utara, Medan 20155, Indonesia

**Keywords:** COVID-19, mutated SARS-CoV-2 virus, vaccine, post vaccine surveillance

## Abstract

The ongoing COVID-19 pandemic, as a result of the SARS-CoV-2 virus, since December 2019, is a major health problem and concern worldwide. The pandemic has impacted various fields, from the social to the development of health science and technology. The virus has been mutating and thus producing several new variants, rushing research in the field of molecular biology to develop rapidly to overcome the problems that occur. Vaccine clinical studies are developing promptly with the aim of obtaining vaccines that are effective in suppressing the spread of the virus; however, the development of viral mutations raises concerns about the decreasing effectiveness of the resulting vaccine, which also results in the need for more in-depth studies. There have been 330 vaccines developed, including 136 clinical developments and 194 pre-clinical developments. The SARS-CoV-2 variant continues to evolve today, and it poses a challenge in testing the effectiveness of existing vaccines. This is a narrative review describing the emergence of the COVID-19 pandemic, development of vaccine platforms, identification of concerning mutations and virus variants in various countries of the world, and real-world monitoring of post-vaccination effectiveness and surveillance.

## 1. Introduction

Severe acute respiratory syndrome coronavirus-2 (SARS-CoV-2) is a highly contagious virus that emerged at the end of 2019 and has caused an upper respiratory disease pandemic, currently known as Coronavirus Disease 2019 (COVID-19) [1]. On 11 March 2020, WHO declared COVID-19 a global pandemic, and it was reported that COVID-19 had spread in 197 countries (on 25 March 2020) with a mortality rate of 18,440 and a recovery rate of 114,802 globally [2]. Efforts to defeat the COVID-19 pandemic continue to date, and vaccinations have been implemented globally.

Until now, the SARS-CoV-2 virus continues to mutate, and there are several variants that have become a variant of concern (VOC), including B.1.1.7 (Alpha), B.1.351 (Beta), P.1 (Gamma), and B.1.617.2 (Delta). At the end of 2020, new variants emerged, namely C.37 (Lambda) and B.1.621 (Mu), which were included in the Variants of Interest (VOI) category [3]. The Alpha variant is estimated to have a 61% (42–82%) higher risk of death than the pre-existing variants, with greater infectiousness and disease severity [4]. P.1, another highly contagious variant, has been circulating in Brazil since the middle of 2020. This variant has been linked to an outbreak of infections in Manaus, Brazil’s Amazon, which has put the healthcare system on the verge of collapsing. B.1.351 was discovered late last year in South Africa [5], while the Delta variant is known to be notoriously contagious. Within five weeks of its discovery in April–June 2021, the Delta variant became the dominant SARS-CoV-2 variant in Mesa County, Colorado, and is now the dominant variant in the United States [6]. Research to find vaccines, clinical trials, and vaccinations on a large scale to defeat the SARS-CoV-2 virus have been carried out to date, but many questions are still unanswered, including: What is the effectiveness and efficacy of the COVID-19 vaccine? Is the vaccine effective against the many SARS-CoV-2 variants? This narrative review has been prepared to answer these questions using searched and collected data on the vaccines of SARS-CoV-2 mutated virus and post-vaccination surveillance.

## 2. Methods

We used prominent search engines, namely Google Scholar, PubMed, Scopus, and BMC. The search keywords were effective and efficacy vaccine, mutated SARS-CoV-2 virus, COVID-19 vaccine variant, post-vaccination, and surveillance strategy. After the search was complete, and all duplicates were removed, then the abstracts of the remaining articles were reviewed to ensure they address the review question. Table 1 contains the inclusion criteria that were used to establish the study’s relevance. The authors appraised, evaluated, and interpreted the selected articles. This narrative is reflected in the literature search (e.g., years considered, language, publication status, study design, and databases of coverage). We summarized and synthesized the findings from the selected articles and integrated them into the narrative review.

**Figure 1:**
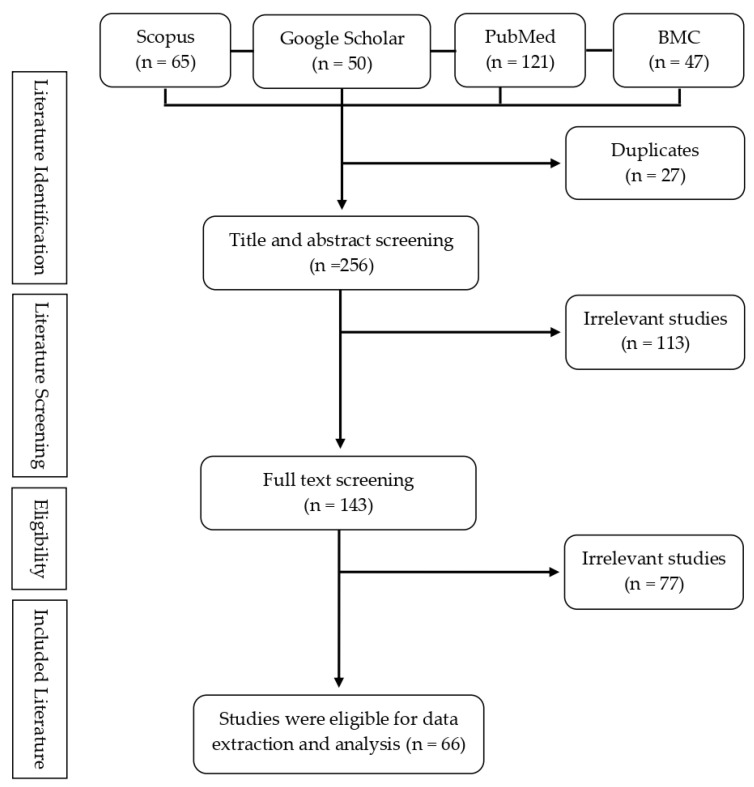
The PRISMA flow diagram depicts the process of selecting eligible studies.

Figure 1 illustrates the PRISMA flow diagram for publication screening. The literature search described in Table 1 resulted in the discovery of 283 publications at first. The literature screening, which consisted of title, abstract, and full-text screenings, yielded 66 final publications based on the relevance of the initial papers to the established research topics for this study. Overall, only 25% of the publications initially identified in PubMed, Scopus, Google Scholar, and BMC publication databases were included in the screening processes. The approximate publications that were recorded out of publications with duplicates were indicated by the literature search results from major scholar databases such as PubMed and Scopus (Figure 1).

## 3. SARS-CoV-2 and Receptors

SARS-CoV-2 is a subfamily of Coronaviridae (CoV), part of the Coronaviridae RNA virus family. The two types of CoV viruses that have caused serious illness are SARS-CoV-1 that caused SARS in 2002–2003, and MERS-CoV causing MERS that occurred in the Middle East in 2012 [7]. SARS-CoV-2 was first identified by independent Chinese scientists through the bronchoalveolar lavage fluid of a patient who had severe pneumonia at the start of a COVID-19 case in Wuhan [1]. The identification results presented the presence of beta-coronavirus that showed 85% genomic similarity with the Bat-SARS-like CoV virus (bat-SL-CoVZC45, GenBank: MG772933.1); afterward, the virus was isolated and named 2019-nCoV. The electron micrograph from 2019-CoV shows the sphere shape with various diameters between 60–140 nm, with a clear spike between 9–12 nm in size; thus, it looks like a solar corona [8].

**Figure 2:**
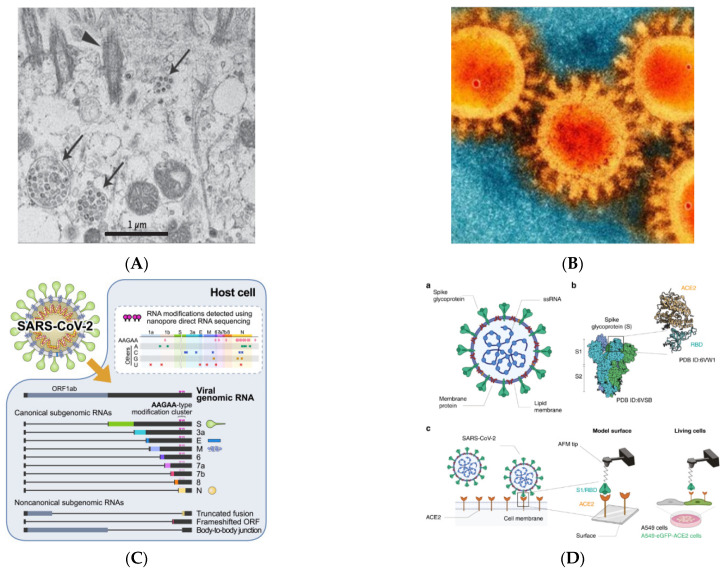
Visualization of SARS-CoV-2 (adapted with permission from Zhu et al., 2019; Bharadwaj et al., 2020; Kim et al., 2020 and Yang et al., 2020) [8,9,10,11] (**A**) Visualization of SARS-CoV-2 with Transmission Electron Microscopy (Virus particles in the human airway epithelial cell). Arrowheads represent extracellular virus particles, while arrows represent inclusion bodies generated by virus components; (**B**) SEM image of SARS-CoV-2 (Large protrusions emerged out from the spike of viral surface, which forms a crown-like appearance, that’s why given the name ‘coronavirus’ (Latin word means crown)); (**C**) Transcriptomic (The full-length genomic RNA and nine major subgenomic RNAs); (**D**) SARS-CoV-2—ACE2 interaction (The initial attachment of SARS-CoV-2 to cells involves specific binding between the viral S glycoprotein and the cellular receptor, ACE2. The interactions are monitored by AFM on model surfaces, where the ACE2 receptor is attached to a surface and the S1 subunit of the RBD onto the AFM tip, and on A549 living cells expressing or not fluorescently labeled ACE2). a. A SARS-CoV-2 particle, an enveloped ssRNA virus with the spike glycoprotein (S) on its surface that mediates binding to host cells, is depicted schematically. b. A complex between the receptor-binding domain (RBD, a subunit of the S glycoprotein) and the ACE2 receptor has previously been discovered by structural research. c. A diagram depicting the use of AFM to investigate SARS-CoV-2 binding.

The SARS-CoV-2 spike protein is 1273 amino acids long, a little longer than SARS-CoV (1255 amino acids) and Bat-SARS-CoV (1245–1269 amino acids). The China National Center for Bioinformation’s 2019 Novel Coronavirus Resource identified a variant of the SARS-CoV-2 strain, of which 15,018 mutations were discovered worldwide [1]. Visualization of SARS-CoV-2 with transmission electron microscopy exhibited the transcriptomics of SARS-CoV-2, presented in Figure 2A–C [9].

SARS-CoV-2 shares a 79 percent genome sequence similarity with SARS-CoV, and a 50 percent similarity with MERS-CoV. Its genome organization is similar to that of other beta-coronaviruses. The six functional open reading frames (ORFs) are arranged from 5′ to 3′: replicase (ORF1a/ORF1b), spike (S), envelope (E), membrane (M), and nucleocapsid (N). The majority of the proteins encoded by SARS-CoV-2 are similar in length to the corresponding proteins in SARS-CoV. Except for the S gene, which differs, SARS-CoV-2 shares more than 90% amino acid identity with SARS-CoV [1]. The replicase S gene encodes a huge polyprotein (pp1ab) that is proteolytically degraded into 16 non-structural proteins involved in transcription and viral replication. The majority of these non-structural SARS-CoV-2 proteins share more than 85% of their amino acid sequence with SARS- CoV-2 [1].

SARS-CoV-2, like SARS-CoV, binds to the human angiotensin-converting enzyme 2 (ACE2) receptor. ACE-2 is a membrane receptor protein that is primarily found in adipose tissue, the kidney, the heart, and the small intestine. In humans, SARS-CoV-2 infection causes a range of symptoms from mild to severe respiratory failure. When SARS-CoV-2 binds to airway epithelial cells, it replicates, migrates, and enters alveolar epithelial cells in the lungs. This rapid replication causes a strong immune response known as a cytokine storm, which results in acute respiratory distress syndrome and respiratory failure, which are the leading causes of death in COVID-19 patients [12,13,14]. The upper respiratory tract provides the first line of defense in that it activates a very complex system of signaling and recruitment of immune cells in order to prevent pathogens from reaching the alveoli, the most important part of the respiratory system, as they are responsible for gas exchange. Nevertheless, SARS-CoV-2 or other coronavirus are able to reduce or delay the expression of cytokines in human lung epithelial cell lines. This is an effective system to escape immune recognition by innate receptors in the infected cell, which could therefore facilitate the progression of the virus into alveolar epithelial cells [15].

Previous research has used the force-distance (FD) curve-based atomic force microscopy to investigate the biophysical properties of the SARS-CoV-2 S-glycoprotein binding to ACE2 receptors on model surfaces and in living cells (FD-curve-based AFM). They extracted the kinetics and thermodynamics of the in vitro interactions and compared the binding properties of both the S1 subunit and the RBD. Both have been tested as potent binding inhibitor peptides targeting the viral S glycoprotein, and a significant reduction in binding properties has been observed. Figure 2D depicts the SARS-CoV-2 scheme as well as the binding scheme with the ACE2 receptor [11]. Coronavirus entrance necessitates the virus’s attachment to the ACE2 receptor on the host cell’s surface, followed by priming by TMPRSS2. The possible SARS-CoV-2 cofactors ACE2, TMPRSS2, and FURIN are expressed largely in bronchial cells switching from secretory to ciliated identity, according to this study, which used previously unreported single-cell data from the human lung and bronchia [16].

## 4. Mutation in the SARS-CoV-2

Several variants of SARS-CoV-2 are circulating globally and have been identified, including the B.1.1.7 (Alpha) from the United Kingdom, B.1.351 (Beta) from South Africa, B.1.617.2 (Delta) from India, P.1 (Gamma) from Brazil, and B.1.1.529 (Omicron). The Alpha variant came from the UK and was first identified in December 2020 [17,18]. Although distinct, the Alpha and Beta variants share common characteristics, including known escape mutations discovered in vitro through antibody pressure selection [19]. The SARS-CoV-2 B.1.617.2 Variant of Concern (VOC) or Delta variant, first detected in India, has now displaced the B.1.1.7 (Alpha) strain, which emerged in the UK with the second COVID-19 wave in late 2020. The B.1.617.2 variant may be transmitted at a higher rate than other variants [17,20]. The WHO Technical Advisory Group on SARS-CoV-2 Virus Evolution recognized the B.1.1.529 COVID-19 variant, which was first detected in Botswana and South Africa, as the Omicron variant of concern on 26 November 2021. Omicron, the SARS-CoV-2 variant responsible for a cluster of cases in South Africa and that is now spreading around the world, is the most heavily mutated variant to emerge so far and carries mutations similar to changes seen in previous variants of concern associated with enhanced transmissibility and partial resistance to vaccine-induced immunity [21].

The WHO and the NIAID directors conveyed to the public and health practitioners about a new variant called “Mu” in the COVID Briefing on 2 September 2021. The Mu-variant is a SARS-CoV-2 variant named B.1.621, according to PANGO lineage, which was first identified in a sample of 11 January 2021 in Colombia [22]. The status of Mu-variant in the world is designated as a VOI by WHO on 30 August 2021. According to WHO data, the distribution of Mu-variant in the world as of 1 September 2021 has been found in at least 39 countries [3]. The Mu-variant carried at least 21 point mutations in the genetic material of SARS-CoV-2, of which nine were in the viral spike protein. This variant carries several key mutations previously known in other variants. The key mutation carried by the Mu-variant is N501Y (as in the Alpha-variant); E484K (as in the Beta-variant); P681H (as in the Delta-variant) [23].

WHO decided to monitor this variant because the combination of mutations carried by the Mu-variant has the potential to decrease antibody neutralization, as shown in early studies of convalescent plasma and vaccine sera (however, the scientists from the Virus Evolution Working Group agree that further research is needed). Its global prevalence continues to decline (currently < 0.1%); however, this variant is still under scrutiny due to its consistent development in Ecuador and Colombia [3,24]. The WHO Virus Evolution Working Group (now called the Technical Advisory Group on Virus Evolution) released recent enhancing readiness for Omicron (B.1.1.529): technical brief and priority actions for WHO members on 10 December 2021. It has been mentioned that the current understanding of the Omicron variant based on recent data is likely to evolve as more data becomes available.

## 5. Types of COVID-19 Vaccines

**Figure 3:**
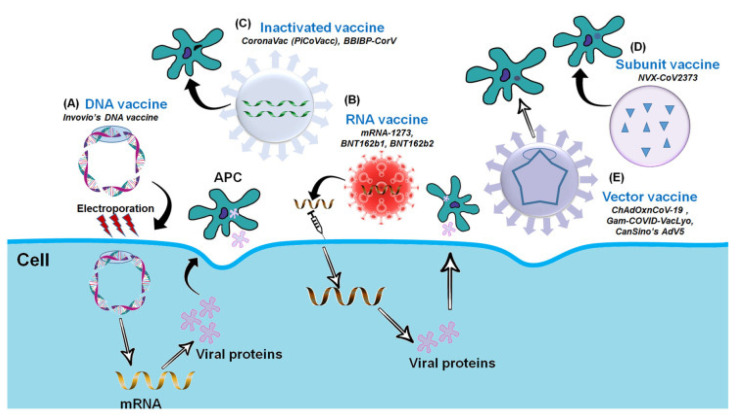
The Platform for COVID-19 Vaccine (adapted with permission from Ashraf et al., 2021) [27]. Platforms for the COVID-19 Vaccine Development. (**A**) DNA vaccine: Plasmid DNA expressing S protein. (**B**) RNA vaccine: mRNA-based (RBD of S-protein). (**C**) Inactivated vaccine: Inactivated whole SARS-CoV-2. (**D**) Subunit vaccine: Recombinant S-protein and (**E**) Vector-based vaccine: Replicating or Non-replicating viral vector used for the delivery and expression of S protein.

Over the last few decades, advances in molecular biology and vaccinology have resulted in the development of a diverse range of novel vaccine platform technologies. These platform technologies include live pathogen inactivation and targeted attenuation to the delivery of synthetic peptide antigens and recombinant protein antigens, as well as virus-like particles (VLPs), non-replicating and replicating viral vectors, polysaccharide-protein conjugates, and nucleic acid-based (DNA and RNA) vaccines. Many of the currently marketed vaccines against infectious diseases are based on these platform technologies [25,26]. The COVID-19 Vaccine Development Platform consists of the following components: inactivated whole SARS-CoV-2, DNA-based vaccine (plasmid DNA expressing S protein), m-RNA-based vaccine (receptor binding domain of spike protein), subunit vaccine (recombinant spike protein), and vector-based vaccine (replicating or non-replicating viral vector used for the delivery of spike protein). Figure 3 depicts the COVID-19 Vaccine Development Platform [27].

Based on data from WHO as of 7 December 2021, there are currently 330 COVID-19 vaccines under development, with 136 vaccine candidates having entered the clinical-phase trial and 194 others still in the pre-clinical phase. Among the vaccine candidate platforms currently being tested are protein subunit 47 (35%), viral vector non-replicating 20 (15%) and replicating 2 (1%), DNA based vaccines 15 (11%), mRNA based vaccines 22 (16%), and inactivated virus 13 (13%). According to the 136 types of vaccines that have been clinically tested, 10 vaccines have reached stage 4 clinical trials [28].

### 5.1. Inactivated Virus-Based Vaccines

Pre-clinical studies have been conducted on a number of COVID-19 vaccine candidates based on well-established technology. This method is based on an existing technology platform for pathogen inactivation in blood products, in which ultraviolet light and riboflavin are used to inactivate the virus through targeted damage to nucleic acids, while proteins and viral antigens are preserved. This technique has been shown to be effective in inactivating MERS-CoV. The development of conventional inactivated vaccines necessitates the cultivation of high titers of infectious virus, which, in the case of SARS-CoV-2, must take place in biosafety level 3 facilities, posing a major safety concern. Furthermore, incomplete virus inactivation poses a potential risk to vaccine production workers, as well as the possibility of disease outbreaks in vaccinated populations and the induction of harmful immune or inflammatory responses [26,29].

CoronaVac is a vaccine with an inactivated virus-based platform. This vaccine has varying efficacy, with the latest efficacy results from phase 3 clinical trials in Indonesia showing this vaccine is quite effective with 65.3% efficacy after the second dose of 1620 participants aged 18–59 years [30].

### 5.2. DNA-Based Vaccines

The introduction of the DNA vaccine, which encodes for the antigen and an adjuvant that induces the adaptive immune response, has been the most revolutionary approach to vaccination. The transfected cells express the transgene, supplying a steady supply of transgene-specific proteins that are very similar to the live virus [31]. In the form of DNA, this vaccination comprises a subset of the virus’s genes. The DNA is employed as a template for in situ expressions of possibly innocuous viral proteins, which trigger a protective immune response after injection. The safety and scalability for large production are two of the most significant advantages of this type of vaccine.

The DNA has to enter the nucleus in order to be transcribed. The worry arises in terms of the potential long-term risk of tumorigenicity, especially when injected into young people. These concerns are supported by a document issued by the FDA under the title “Long-Term Follow-up after Administration of Human Gene Therapy Products, Guidance for Industry” [32]. In contrast, a 1997 study found that parenteral administration of hybridoma DNA does not result in the development of local tumors. Furthermore, there was no evidence of the hybridoma DNA’s systemic carcinogenic potential [33]. Behind the conflicting DNA-based vaccine platforms, there are several vaccines being developed, including INO-480+electroporation, nCov vaccine, AG0301-COVID19, and GX-19N [28].

### 5.3. mRNA-Based Vaccines

RNA vaccines contain virus genes in the form of mRNA, which is then translated into viral proteins after cytosolic delivery. The mRNA is a new, non-infectious, and non-integrating platform with almost no risk of insertional mutagenesis. Currently, non-replicating RNA and virus-derived self-replicating RNAs are being studied. The immunogenicity of the mRNA can be reduced, and changes can be made to improve the stability of these vaccines [26,31].

The mRNA-1273 vaccine, developed by Moderna Therapeutics (Cambridge, MA, USA) and the National Institute of Allergy and Infectious Disease (NIAID), was the first candidate vaccine to enter Phase I clinical testing, just 42 days after the full SARS-CoV-2 genome was sequenced (ClinicalTrials.gov identifier NCT04283461) [34,35].

Recent studies of the BNT162b2 vaccine have shown that after 6 months of use, the vaccine is safe and has acceptable adverse events. The efficacy of this vaccine reaches 91.3% and has a high efficacy advantage in dealing with beta variants [36]. In phase 4 clinical trials, the vaccine was tested as a booster or third dose in participants aged over 16 years and patients with multiple sclerosis [37,38,39].

### 5.4. Protein Subunit-Based Vaccine

Vaccines based on synthetic peptides or recombinant antigenic proteins are known as subunit vaccines. Subunit vaccines have low immunogenicity and require adjuvant support to enhance the vaccine-induced immune response. Subunit vaccines contain only specific viral antigenic fragments and no additional pathogenic virus components. As a result, this method eliminates concerns about incomplete viral inactivation, virulence recovery, and pre-existing anti-vector immunity. As a result, subunit vaccines are widely regarded as extremely safe. Furthermore, subunit vaccines can specifically target well-characterized neutralizing antigenic epitopes and improve immunogenicity and/or efficacy when combined with adjuvants. Adjuvants can extend the antigenic material’s biological half-life or improve the immunomodulatory cytokine response [26].

Vaccines developed with this model include NVX-CoV2373 (Novavax, Inc.). In an animal model, it demonstrated high immunogenicity by measuring anti-spike antibodies, which prevent the spike protein from attaching to the receptor, as well as wild-type virus-neutralizing antibodies [31]. Adult participants were given a two-dose regimen of the NVX-CoV2373 vaccine, which provided 89.7 percent protection against SARS-CoV-2 infection and demonstrated high efficacy against the B.1.1.7 variant [40].

### 5.5. Viral Vector-Based Vaccine

A vaccine based on viral vectors is a promising anti-pathogen treatment. These vaccines are highly specific in terms of delivering genes to target cells, efficient in terms of gene transduction, and, once again, inducing an immune response. They provide a long-term and high level of antigenic protein expression and thus have a high potential for prophylactic use, as these vaccines activate and prime cytotoxic T cells (CTL), resulting in the elimination of virus-infected cells [31,41]. Viral vectors are used to deliver vaccine antigens to target cells or tissues, and both replicating and non-replicating viral vectors are available. Adenoviruses and poxviruses are examples of viral vectors of replicating and non-replicating forms.

One of the viral vector vaccine platforms that have been used today is ChAcOx1 nCoV-19 (AstraZeneca and Oxford). Based on clinical trial data from 17,177 people consisting of 8948 in the UK, 6753 in Brazil, and 1476 in South Africa, showed high safety and efficacy in participants aged 18–55 years [42].

## 6. Efficacy and Effectiveness of COVID-19 Vaccines

To determine the effectiveness and efficacy of the vaccine against the SARS-CoV-2 mutant, Weissman and colleagues conducted experiments using the serum of mice, non-human primates, and humans. Experiments were carried out using a pseudovirus containing spike D614 or G614. The results showed that mice, non-human primates, and humans vaccinated using the mRNA-LNP platform produced an antibody response that not only recognized the G614 mutation but also had stronger neutralization titers against this viral variant [43,44,45].

The study used a pseudovirus that expresses a wild-type spike protein or a mutated spike protein containing eight amino acid changes, detected in the variant B. 1.1.7, to assess the immune response of individuals after vaccination with the BNT162b22 mRNA-based vaccine by measuring the neutralizing antibody response after the first and second immunization. Individuals who received the vaccine had variable neutralizing titers against the wild-type pseudovirus in their blood, which were slightly lower against variation B.1.1.7. Some individuals who had recovered from COVID-19 showed this drop in their serum as well [46].

Another Brazilian variation, P.1, was found to be resistant to not only some neutralizing monoclonal antibodies but also to neutralization by convalescent plasma and vaccination serum in a prior investigation. With the pseudovirus and native P.1 virus, the level of resistance was greater for monoclonal antibodies than for vaccine serum. The P.1 trimer only assumes a conformation in which one of the receptor-binding domains is in the “up” position, which is known to enhance binding to the ACE2 entry receptor, according to the cryoelectron microscopy structure of the soluble prefusion stabilized spike. Local rather than global conformational alterations appear to be the source of the P.1 mutation’s functional impact. Current antibody therapy is threatened by the P.1 mutation, but vaccination efficacy is limited [47,48].

Furthermore, a study was carried out to see if monoclonal antibodies, convalescent serum, and vaccinations might neutralize B.1.617.1 and B.1.617.2, as well as a structural analysis of the Fab/receptor-binding domain (RBD) complex and a map of the antigenic space of variant flows. When compared to the Wuhan-associated ancestor strain, neutralization of both viruses was reduced, but there was no evidence of broad antibody release, as reported in B.1.351. However, serum B.1.351 and P.1 showed a greater drop in B.1.617.2 neutralization, suggesting that people who have been infected with this variety before may be more susceptible to reinfection with B.1.617.2 [49]. Although it has 12 mutations in its spike protein relative to the wild type SARS-CoV-2, which was first detected in Wuhan, China, in December 2019, B.1.617.2 lacks mutations at amino acid positions 501 or 484 in its ACE2 receptor-binding domain, which is commonly associated with VOCs or escapes from neutralizing antibodies (NAbs) [50]. Previous studies have shown an effective vaccine against the Delta-variant in the world. A program called UK-REACT-1, led by the Imperial College London team, tested more than 100,000 volunteers in the UK every few weeks. Ct analysis from the PCR was carried out for samples from May–July 2021, when the Delta-variant became dominant in the UK. Volunteers were tested by PCR as well as sequential genome sequencing to confirm confirmed SARS-CoV-2 infections and the Delta-variant in infected individuals. Paul Elliot, one of the members of the research team, noted the strength of the study because it took a random sample of the population and included people who tested positive but were asymptomatic. The results of a large-scale study exhibit among individuals who tested positive for COVID-19 showed the following [51,52]:

(1) Vaccinated individuals are estimated to have lower viral loads compared to the unvaccinated;
(2) Vaccinated individuals exhibit milder symptoms compared to unvaccinated ones;
(3) The UK implemented a fairly rapid vaccination program to protect the population (currently, it has reached 70%–80% of the total population, with vaccines used including Pfizer, Moderna, AstraZeneca, and Johnson & Johnson).

Vaccine platforms that have been developed and passed phase 3 clinical trials and have been used to overcome the COVID-19 pandemic in various countries, including Pfizer-BioNTech, Moderna, AstraZeneca-Oxford, Janssen, Sputnik V, CanSino, Sinovac, and Sinopharm. Table 2 contains data on its efficacy and effectiveness in combating the COVID-19 pandemic and its variants, whereas Table 3 displays data on its effectiveness against severe disease.

Based on the data collected in the Table 2 and Table 3, it is known that various variants of SARS-CoV-2 are able to reduce the effectiveness of several vaccines that have received approval. However, vaccination can increase the severity of the case-fatality reduction. Not all vaccines have published data regarding their effectiveness and efficacy against various VoCs. The efficacy and effectiveness of vaccination in a pandemic is not only influenced by the infecting variant, but internal factors such as age, comorbidities, and even race, have the possibility of influencing these numbers. This is an opportunity to publicize the real conditions experienced in health care facilities along with the mass vaccination process that is still being carried out in various parts of the world.

## 7. Post-Vaccination Surveillance Strategy

Post-vaccination monitoring needs to be carried out considering the vaccines used have not been completed in clinical trials and the limited number of participants in clinical trials. In practice, several problems were reported, such as death from blood clots after vaccination and other serious effects. Post-vaccination monitoring can be done using two pharmacology approaches, namely pharmacovigilance and pharmacoepidemiology. Pharmacovigilance, also known as drug safety surveillance, is primarily concerned with the timely identification of novel adverse drug reactions (ADRs) that are distinct in clinical nature, severity, and/or frequency. Pharmacoepidemiology is the population-based study of drug use and the risks associated with it. The importance of using pharmacovigilance should be emphasized by highlighting that the life of a drug truly begins after marketing [66,67].

The FDA Adverse Event Reporting System (FAERS) was created in the United States to gather ADRs from healthcare providers, patients, and pharmaceutical corporations. FAERS intends to support pharmaceutical and biological product post-marketing surveillance initiatives [68,69]. In Europe, the European Medicines Agency (EMA) established the Pharmacovigilance Risk Assessment Committee, which is in charge of all aspects of medicine safety assessment and monitoring. In the EudraVigilance database, adverse events are noted, recorded, and analyzed, and it is regarded as one of the world’s largest databases, with over 16.7 million individual case safety reports (ICSRs) [70].

Besides, in Indonesia, the Food and Drug Supervisory Agency (*Badan Pengawas Obat dan Makanan*/BPOM) monitors the safety, efficacy, and quality of COVID-19 vaccines. In order to guarantee requirements compliance, the BPOM continues to supervise the implementation of clinical trials, including evaluation process accelerate for grant of the Clinical Trial Protocol Approval (*Persetujuan Protokol Uji Klinik*/PPUK), and thereafter inspections ensuring that clinical trials are conducted in accordance with the approved clinical trial protocols, and also regulations implementation of Good Clinical Practice (GCP). Monitoring of the clinical trial subject safety was established by the Health Research Ethics Committee of Padjadjaran University. The BPOM has implemented comprehensive inspections of vaccine production facilities to warrant that manufacturers apply Good Manufacturing Practices (GMP) standards throughout the vaccine manufacturing process, consistently commence the manufacture of vaccine raw materials (upstream), and thereafter vaccine formulation (downstream), until the process of vial filling for acquiring the end-product. Furthermore, the entire data (safety, efficacy, and quality aspects) is compulsorily submitted by the pharmaceutical industry to the BPOM for an evaluation process that refers to national and international evaluation guideline standards. The evaluation process is conducted via conferences with the National Committee for Drug Evaluation, experts, and clinicians from the Indonesian Doctors Association (*Ikatan Dokter Indoensia*/IDI). Specifically for vaccines, conferences have been organized with the Indonesian Technical Advisory Group on Immunization (ITAGI) [71].

At the international level, the WHO Uppsala Monitoring Center (UMC) maintains a large database of ADR reports known as “VigiBase” [72]. VigiBase is the WHO’s one-of-a-kind global database of individual case safety reports (ICSRs). It is the world’s largest database of its kind, with over 25 million reports of suspected adverse effects of medicines submitted by member countries of the WHO Program for International Drug Monitoring since 1968. It is constantly updated with new reports [73]. In addition, Ontario will analyze the effectiveness of COVID-19 vaccines against a variety of outcomes and subgroups of interest using linked data at ICES (e.g., age group and comorbidities). The goal of this study is to determine how effective immunizations are against laboratory-confirmed COVID-19 infection (symptomatic and asymptomatic) as well as clinical severity assessments, including hospitalization and mortality. Vaccine efficacy (VE) assessments will also look at how successful the vaccine is after one and two doses, as well as VE against SARS-CoV-2 subtypes (i.e., variants of concern) [74]. It is critical to evaluate the performance of COVID-19 vaccines as part of post-implementation surveillance in the context of “real world” program implementation, which includes immunization of some groups excluded from trials (e.g., LTC residents, immunosuppressed, and pregnant individuals) and the use of an extended second dose interval (with some exceptions) [74].

Several vaccine effectiveness studies in the United Kingdom (UK) have found that two doses of COVID-19 with the Delta variant vaccination are between 65 and 95 percent effective at avoiding symptomatic disease, with higher levels of protection against severe disease, including hospitalization and death. Protection against infection and symptomatic disease appears to wane over time; however, protection against severe disease remains high in most groups at least 5 months after the second dose. Vaccine coverage, proof of vaccine effectiveness, and the most recent COVID-19 disease surveillance indicators are used to analyze the vaccination program’s effects on the population. Vaccine coverage indicates the percentage of people who have received one or two doses of COVID-19 vaccine. By 10 October 2021, 65.5% of people in England had received one dose of the vaccine, and 60.4% had received two doses of the vaccine. According to the program’s launch, the oldest age groups have the most coverage. COVID-19 cases, hospitalizations, and deaths are broken down by vaccination status. According to blood donor antibody testing, 98.0% of adults now have antibodies to COVID-19 from infection or vaccination, compared to 18.7% who have antibodies from infection alone. The effectiveness of vaccines is determined by comparing disease rates in vaccinated and unvaccinated people [75]. The summary of the latest real-world information on vaccine effectiveness from research in UK populations (concentrating on information about the Delta variety, which is currently the most common in the UK) is shown in Table 4 as well [75].

Phase 3 vaccine studies are conducted in well-defined populations that may exclude some categories, such as immunocompromised people, pregnant women, and children. These tests are carried out in optimal settings, with excellent cold chain storage and maintenance, and the recommended interval between dosages is strictly followed. It is crucial to assess effectiveness as it is used in the real world, as this may differ from clinical trial efficacy. Clinical studies are usually powered for a primary goal of virologically verified symptomatic disease within a short follow-up time so that efficacious vaccinations can be introduced as quickly as possible. Understanding the effectiveness against different endpoints (such as disease severity and onward transmission), effectiveness in different subgroups of the population, and duration of effectiveness are all important factors in deciding which vaccines to use as the program evolves, whom to offer them to, and whether booster doses are necessary. Using a screening and test-negative case control method, the expanded surveillance will be utilized to monitor age-specific vaccine effectiveness in targeted populations, as well as identify risk factors for and outcomes of vaccine failure [76].

## Figures and Tables

**Table 1:** List of questions used for studies inclusion and exclusion during combined title and abstract, and full-text screenings.

Screening Stages	Questions	Screening Outcome
Title and abstract screening	Does the study focus on SARS-CoV-2? Does the study focus on mutation in the SARS-CoV-2? Does the study present COVID-19 vaccines?	Studies are included if they satisfy all questions
Full-text screening	Do the study present effective or efficacy vaccines? Does the study contain vaccines containing a percent of efficacy? Does the study provide the effectiveness of vaccines to reduce infection? Does the study present the effectiveness of vaccines to reduce severe/death? For included studies, additional following open questions are given to identify general information of the studies: What is the percent of effectiveness and efficacy of a vaccine? What is the post-vaccination surveillance strategy? Publication type (e.g., J = journal article, P = Proceeding Conference, T = thesis, B = Book chapter, R = Report) What is the type of study? (L: lab measurement, R: review, O: Opinion, or P: preprint)	Studies are included if they satisfy at least two screening questions

**Table 2:** Efficacy and effectiveness of COVID-19 vaccines that have received emergency use authorization against various SARS-CoV-2 variants [28,53,54,55,56].

Vaccine Platform	Vaccine Developers	Approval Status (Number of Countries)	Efficacy (%) (Number of Participants)	Effectiveness to Reduce Infection (%)			
				Alpha	Beta	Delta	Gamma
Inactivated Virus-Based Vaccine	Sinovac (CoronaVac)	49	82.22–84.44 (18–59 years old) and 62.69–70.37 (>60 years old) from 434 participants [57]; 83.5 from 10,214 participants (6646 vaccinated, 3470 placebo) [58]; 65.30 (1620 participants) [30]	n/a	65.9 [54]	n/a	65.9 [54]
	Sinopharm (BBIBP-CorV)	77	78.1 (40,382 participats) [59]	–	–	–	–
mRNA-Based Vaccine	Pfizer-BioNTech (BNT162b2)	113	95 (18,198 vaccinated, 18,325 Placebo) [60]	89.5 (50/19,354 vaccinated/unvaccinated PCR-positive) and (465/15,939 vaccinated/unvaccinated PCR-negative) [55]	75 (179/19,396 vaccinated/unvaccinated PCR-positive) and (698/18,877 vaccinated/unvaccinated PCR-negative) [55]	88 (122 case/15,749 control) after second dose [56]	85 [54]
	Moderna (mRNA-1273)	77	94.1 (15,181 vaccinated, 15,170 placebo) [61]	91 [54]	78 [54]	70 [54]	78 [54]
Viral Vector-Based Vaccine	AstraZeneca-Oxford (ChAdOx1-S)	172	70.4 (5807 vaccinated, 5829 placebo) [62]	74.5 (94 case/8244 control) after second dose [56]	75.4 (3/944 vaccinated, 12/938 placebo) [63]	67.0 (218 case/8244 control) after second dose [56]	<70 [54]
	Janssen (Ad26.COV2.S)	79	66.9 (19,630 vaccinated, 19,691 placebo) [64]	–	52 [54]	–	52 [54]
	Gamaleya (Sputnik V)	74	91.6 (14,964 vaccinated, 4902 placebo) [65]	–	–	–	–
	CanSino (Convidecia)	9	65.7 [53]	–	–	–	–
Protein Sub-unit-Based Vaccine	Novavax (NVX-CoV-2373)	Not Approved	89.7 (15,187 participant) [40]	86.3 (15,187 participant) [40]	60 [54]	60 [54]	60 [54]

**Table 3:** Effectiveness of COVID-19 vaccines against severe disease [54,55].

Vaccine Developers	Effectiveness to Reduce Severe Disease/Death (%)							
	Alpha	Participants	Beta	Participants	Delta	Participants	**Gamma**	**Participants**
Sinovac (CoronaVac)	n/a	n/a	87.5	n/a	n/a	n/a	87.5	n/a
Pfizer-BioNTech (BNT162b2)	54.1 (after one dose) [55] 100.0 (after second dose) [55]	PCR-positive: 30 vaccinated/468 unvaccinated PCR-negative: 61/437 PCR-positive: 0/401 PCR negative: 20/381	100.0 (after second dose) [55]	PCR-positive: 0/300 PCR-negative: 14/246	95	n/a	98	n/a
Moderna (mRNA-1273)	94	n/a	94	n/a	96	n/a	94	n/a
AstraZeneca-Oxford (ChAdOx1-S)	95	n/a	n/a	n/a	95	n/a	n/a	n/a
Janssen (Ad26.COV2.S)	n/a	n/a	65–66 (hospitalization) 91–95 (mortality)	n/a	71	n/a	65–66 (hospitalization) 91–95 (mortality)	n/a

**Table 4:** Evidence on the effectiveness of the vaccine against various outcomes Delta variant.

Outcome	Vaccine Effectiveness *		
	Pfizer-BioNTech Cominarty	AstraZeneca Vaxzevria	Moderna Spikevax
Infection	75–85%	60–70%	–
Symptomatic disease	80–90%	65–75%	90–99%
Hospitalization	95–99%	90–99%	95–99%
Mortality	90–99%	90–95%	–

* Estimates of initial vaccine efficacy in the general population following a two-dose course. This is usually true for the first 3 to 4 months after vaccination. Beyond this point, the effectiveness of some outcomes may begin to wane.

## Data Availability

No additional data is available for this paper.
